# Health Effects Related to Wind Turbine Sound: An Update

**DOI:** 10.3390/ijerph18179133

**Published:** 2021-08-30

**Authors:** Irene van Kamp, Frits van den Berg

**Affiliations:** 1 National Institute for Public Health and the Environment, 3721 MA Bilthoven, The Netherlands; 2 Mundonovo Sound Research, 9953 PH Baflo, The Netherlands; frits@mundonovo.nl

**Keywords:** wind turbine, wind farm, rhythmic sound, low-frequency sound, infrasound, health effects, annoyance, sleep disturbance

## Abstract

Commissioned by the Swiss Federal Office for the Environment, an update of an earlier narrative review was prepared for the literature published between 2017 and mid-2020 about the effects of wind turbine sound on the health of local residents. Specific attention was hereby given to the health effects of low-frequency sound and infrasound. The Netherlands Institute for Public Health and the Environment and Mundonovo sound research collected the scientific literature on the effect of wind turbines on annoyance, sleep disturbance, cardiovascular disease, and metabolic effects, as well as mental and cognitive impacts. It also investigated what is known about annoyance from visual aspects of wind turbines and other non-acoustic factors, such as the local decision-making process. From the literature study, annoyance again came forward as the most important consequence of sound: the louder the sound (in dB) of wind turbines, the stronger the annoyance response was. The literature did not show that “low-frequency sound” (sound with a low pitch) results in extra annoyance on top of normal sound. Results of scientific research for other health effects are either not available or inconsistent, and we can conclude that a clear association with wind turbine related sound levels cannot be confirmed. There is evidence that long-term effects are related to the annoyance people experience. These results confirm earlier conclusions. There is increasing evidence that annoyance is lower when people can participate in the siting process. Worries of residents should be addressed in an early stage, by involving them in the process of planning and decision making.

## 1. Introduction

This update of a review on the effects of health effects of wind turbine (WT) sound prepared in 2017 [1,2] is commissioned by the Noise and Non-Ionizing Radiation (NIR) Division of the Swiss Federal Office for the Environment (Bundesamt für Umwelt). An updated overview of the conclusions of scientific studies on health effects of sound from WT was requested, again with special attention to infrasound and low-frequency sound (see Section 3.5 for definitions of infrasound and low-frequency sound). All relevant scientific papers published after January 2017 were collected.

The 2017 review concluded that scientific research did not indicate that WT sound can lead to health effects other than noise annoyance and hence whether these are different from those of other environmental sound sources. It was concluded that amplitude modulation and the rhythmic visual character are characteristic for WT related exposures. Evidence on the effect of night-time WT sound level on sleep was inconclusive. For other health effects, no immediate association with WT sound levels was confirmed. An association between self-reported sleep disturbance and annoyance from WT sound was shown to be consistent. The moderate effect of WT sound levels on community response and the range of other factors influencing this imply that considering other factors associated with community response will benefit mitigating measures. Next to sound, physical and personal aspects, and social aspects such as the circumstances around decision making and siting of a wind farm, communication, and the relation between different parties involved came forward as important factors. Physical aspects of importance include visual aspects, (mis-)match with the landscape, shadow casting, and blinking lights and no effect of light flicker from the blades, vibrations, and electromagnetic fields.

## 2. Materials and Methods

### 2.1. Data Sources and Search

**Figure 1:**
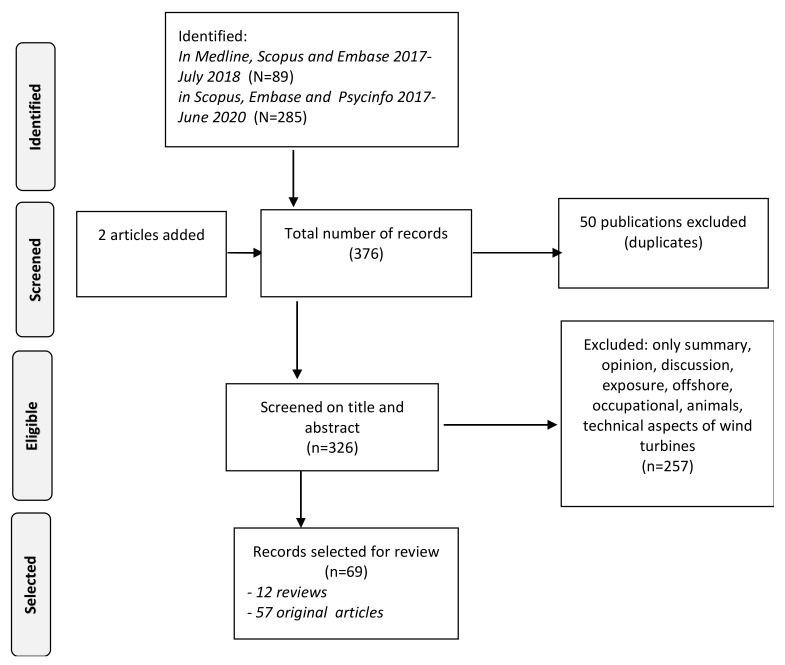
Flowchart of selection process.

This paper summarizes the present knowledge available about the association between WT sound, including low-frequency sound, infrasound, and health. The 2017 review was based on a systematic literature search over the period from 2000 to early 2017. Three databases were searched: Scopus, Medline and Embase. This updated review uses publications between 2017 and June 2020. For the period until July 2018, the same search strategy and databases were used as in 2017. For the second period, until July 2020, the Medline and Embase databases were no longer available, or not in the same format. The platform and search syntax were changed, and a new search strategy was therefore applied. We also added the database PsycINFO. As Figure 1 shows, the literature searches yielded 12 reviews and 57 original papers after reading the full papers, using the criteria described in Section 2.2. This updated systematic review follows a PRISMA based strategy. 

### 2.2. In- and Exclusion Criteria

Observational and experimental studies described in the peer review literature were selected. The search strategy is described in Supplementary Materials (S1). Language was restricted to German, English, French and Dutch. Only studies were included that were published or accepted in peer-review journals or conference proceedings and scientific reports. Studies (quantitative and qualitative) could address all aspects of sound from WTs and there were no restrictions concerning study design. Conditional was a link with health effects or (social) wellbeing, including annoyance and community response. Papers concerning non-human effect, occupational health and safety, offshore, effects on others than residents and papers concerning commentary, editorial or opinion, letter to editor, errata or discussion between people were excluded. With respect to non-acoustic effects, papers were included that addressed visual aspects such as impact on landscape, movement, horizon pollution, light effects, shadow flicker, safety, vibration, electromagnetic fields, demographic, physical, social and personal factors (noise sensitivity, attitude, effect of participation, co-ownership) (For a definition of these variables we refer to [1,2]. Where in 2017 we spoke of “situational factors” we now use the term “physical factors other than noise” abbreviated to “physical factors”. Demographic features are described separately).

### 2.3. Procedure

Articles were grouped into seven categories: reviews, papers on health effects, offshore, exposure, low-frequency sound, visual aspects, social aspects and papers evaluated as not relevant. All reviews and original studies were included for full paper examination, while offshore studies were a priori excluded, and papers from the other categories were reconsidered after reading the abstracts. All material from the selected literature was read and analysed, and were sometimes excluded, e.g., the study was less relevant than originally thought or when they showed up twice in the references. After dual full examination of the reviews and original papers, a final decision was made about inclusion in this review. A meta-analysis on (part of the) data was not considered in this assignment.

### 2.4. Data Extraction

After reaching consensus, the data were extracted, coded and imported into tables. In case of disagreement, the two authors and librarian discussed the options. The following characteristics of the studies were extracted and coded for each selected study on noise and health:

- Acronym/Author and Year of Publication;
- Country;
- Number of studies/participants;
- Study Design (including sampling strategy);
- Quality;
- Exposure source, characterisation and range;
- Outcome type and ascertainment of the outcome.

For the studies investigating the association with annoyance and sleep disturbance and health effects, the following aspects were extracted:

- Adjustment for possible confounders;
- Direction and strength of reported effect size.

For studies investigating the role of physical, social and personal factors, the type of aspects considered was also included in the tables.

### 2.5. Assessment of Quality of Evidence

For the assessment of the quality of studies concerning WT sound and annoyance, sleep disturbance, health and physical, social and personal factors we used as the criteria of the short and user-friendly instruments of the National Institute of Health (NIH) [3]. Aspects accounted for in reviews were: aims clearly described, in- and exclusion criteria clearly defined, a systematic approach to the search strategy was followed, a dual review of title, abstract, and full paper, appraisal of quality of the studies, and details provided on the individual studies. Only reviews were selected that met these criteria. Aspects accounted for in the original studies were study size and response rate (selective participation), exposure assessment, outcome assessment, and confounding. Ratings were categorised as low, medium or high quality. In view of quality, for cardiovascular and metabolic effects, only case-control or cohort studies were included in the update.

## 3. Results

Next to annoyance and sleep, more recently were cardiovascular effects (ischaemic heart disease (IHD) and myocardial infarction, atrial fibrillation, hypertension and stroke) and metabolic effects (diabetes) studied in people living near wind farms, but no studies on obesity. There were no or limited studies available on the association between WT sound and mental and cognitive effects. Our new search of the literature over the 2017–2020 period yielded 10 reviews and 45 new articles on the association between WT sound and health. Twenty-four were included in the review after reading the full text.

The main results are summarized per health outcome and study design and outcome of the selected studies are discussed in more detail. The term ‘sound’ is used to avoid the a priori implication of a negative meaning of the term noise (‘unwanted sound’). The term ‘noise’ is used when a negative meaning is implied, such as in ‘noise annoyance’. Evidence on the effects of WT sound from recent epidemiological studies at population level and smaller scale laboratory experiments is summarized. New findings concerning the influence of physical, social and personal aspects are reviewed. Finally, the literature on the health effects of sound at low frequencies and infrasound is discussed. A discussion of the findings and an evaluation of the quality and results of the new studies in comparison to previous evidence can be found in Section 4.

### 3.1. Reviews on Wind Turbine Sound and Health

Four of the ten reviews published since 2017 (listed in Table 1), address annoyance as (one of) the main health outcomes. Guski et al. [4] identified four studies on WT sound of cross-sectional design and published before 2015. They were selected for review based on the percentage of highly annoyed (%HA), in response to a standard survey question [5], referring to a particular noise source. It was concluded that evidence was only emerging of low quality and did not justify deriving a reliable generalised exposure effect relation (EEr).

Van Kamp et al. [6,7] identified nine new publications on five studies on WT sound and annoyance that met the inclusion criteria, covering the period between 2015 and the end of 2019. 

The narrative review by Simos et al. [8] pertains to 104 articles on 69 studies, and addresses determinants of annoyance, such as sound, visual aspects, real estate prices and safety. No meta-analysis was performed, and the inclusion criteria of studies were not fully described. The authors concluded that the evidence for an effect is meagre and that we probably deal with a ‘nocebo’ effect: the effect of information and negative expectations lead to aversive effects (rather than the WT sound levels themselves). 

Freiberg et al. [9] reviewed the literature published since 2000 and up to mid-2018. Eighty-four articles passed the screening and the eligibility assessment based on the PRISMA approach and included annoyance and other health outcomes. Multiple cross-sectional studies (43) reported that wind turbine noise is associated with annoyance, moderated by social and personal aspects, such as noise sensitivity (NS), attitude towards wind turbines, or economic benefit. The number of studies increased since 2010 and were mostly conducted in OECD member countries. Eleven lower quality studies found an effect while higher-quality studies did not. Research gaps with respect to annoyance, concern the complex pathways of annoyance via non-acoustic factors, the objective investigation of visual WT features, and the interaction between all WT related exposures. 

Since 2017, four reviews address sleep disturbance. Basner and McGuire [10] report evidence of sufficient strength for self-reported and objective indicators of sleep disturbance from environmental noise in general, while evidence for sleep disturbance from WT sound is only emerging and no EEr is available. This is based on the six studies published between 2000 and 2015 that met the rigid selection criteria used. Meta-analysis was performed for five out of six studies and led to inconclusive results. Results show a non-significant association on the pooled data with an odds ratio of 1.60 (95% CI: 0.86–2.94). Two studies were identified using objective measures (actigraphy) to evaluate sleep disturbance due to WT sound. One small study by Lane et al. [11] and the large study of Michaud et al. [12] concluded there was no significant association between WT sound levels and sleep measured with actigraphy.

An update of studies since 2015 [6,7] identified fourteen new articles on sleep disturbance and WT sound, of which eleven concerned self-reported sleep disturbance and three used physiological and behavioural measures.

The review of Micic et al. [13] focuses on potential mechanisms, rather than current evidence for an association between WT sound and sleep disturbance. An association is plausible via two mechanisms: (1) chronic sleep fragmentation from frequent physiological arousals due to sensory disturbances in sleep and (2) chronic insomnia in individuals with higher sensory acuity or those prone to noise annoyance.

Freiberg et al. [9] identified 19 studies on sleep (2000–mid 2018) meeting their criteria. Most included measures of self-reported sleep disturbance and polysomnographic measures. In the higher quality studies, WT sound was not associated with self-reported nor with physiological- or behavioural-measured sleep disturbance, lower quality studies more often suggesting an association. 

Reviews with respect to other effects than annoyance and sleep [14,15,16,17] will be discussed in Section 3.2.3, Section 3.2.4 and Section 3.2.5.

**Table 1:** Overview of the characteristics of the selected reviews on WT Sound and Health.

Author	Country	Design	Number Studies/Participants	Time Range	Quality of Evidence	Sound Level Exposure Range	Outcome	Effect Size	Confounders
Basner and McGuire, 2018 [10]	USA	Review	6 studies (3815)	2000–2014	Very low—low	Per 10 dB	Self-reported or physiologically and behavioural measured sleep disturbance	OR = 1.60 (95% CI: 0.86–2.94) not significant, highly heterogenous	No
Clark and Paunovic, 2018 [15]	United Kingdom	Review	5 reviews	2000–2014	Low to moderate	Poor, often distance used as proxy	Cognitive effects	No effect	No
Clark and Paunovic, 2018 [16]	United Kingdom	Review	0	2000–2014	Na	Poor, often distance used as proxy	Mental health effects	Not applicable.	No
Clark et al., 2020 [17]	United Kingdom	Review	2 studies	2014–2020	Very low to moderate	Poor, often distance used as proxy	Low birth weight, preterm birth, small for gestational age, wellbeing	No effect	No
Guski et al., 2017 [4]	Germany	Review	4 studies	2000–2014	Low quality of evidence	Per 5 dB	Annoyance	summary correlation r = 0.278; *p* = 0.001; 95% CI = 0.11–0.430).	No
Freiberg et al., 2019 [9]	Germany	Review	84 papers/68 studies	2000–2018	Reporting quality 46%; 21% generalizable	Sound levels over a range of frequencies, shadow flicker, blinking lights, or vibrations.	Range of self-reported complaints; physiological effects		Age, sex, and socioeconomic status in 28%
Micic et al., 2018 [13]	Australia	Review	20 studies	2000–2017	Not mentioned	% time above 39 dB(A)	Self-reported or physiologically and behavioural measures of sleep disturbance		Attitude NS, economic benefit
Simos et al., 2019 [8]	Switzerland	Review	104 papers/67 studies	2000–2019	Not mentioned	Not mentioned	Annoyance and other impacts		No
van Kempen et al., 2018 [14]	Netherlands	Review	3 studies	2000–2014	Very low	Per 5 dB	Hypertension, IHD, Diabetes, Stroke		Age, gender
van Kamp et al., 2020 [6,7]	Netherlands	Review	5 studies	2014–2020	Low to moderate	Measured, modelled Sound Pressure Level C- and A- weighted	Annoyance, sleep, HBP, stroke, diabetes		demographics, NS, attitude, shadow flicker, Amplitude Modulation

### 3.2. Original Studies on WT Sound and Health

Results of the selected original studies are summarized in Table 2. Additional papers outside the time frame of 2017–2020 are included since they were missed in our previous review and considered relevant for this update. Average sound levels at the façade of dwellings are used in most studies. For wind energy, two noise measures are commonly used: the LAeq and Lden. Lden (level day-evening-night) is the average noise level for the day, evening and night period calculated for a whole year, including allowances of respectively 0.5 and 10 dB per diurnal period. Lnight is the average night-time level or LAeq, night without the 10 dB allowance. LAeq is the equivalent noise level, usually over an ‘average’ full day (24 h), and the most often used measure. Lden and Lnight are both based on the LAeq and are therefore strongly related, but not easily compared, e.g., the night limit for wind turbine sound in the Netherlands of 41 Lnight corresponds to 43 dB to 45 dB LAeq.

#### 3.2.1. Annoyance

A cross-sectional study by Klaeboe et al. [18] in Norway included 90 participants with a response rate of 38%. WT sound levels were calculated (37 and 47 dB Lden) and annoyance was measured by the 5-point ISO standard scale (the 5- or 11-point ISO standard scale refers to ISO/TS 15666; in the following text this is referred to as the ISO standard scale). Confounding by attitudes, demographics, visual judgements and NS was accounted for. Noise from WTs was considered more annoying than road traffic noise, with a 17–18 dB higher noise level and within the range of 11–26 dBA as reported by Michaud et al. [19] and Janssen et al. [20]. The role of physical, social and personal factors on annoyance was possibly larger than that of WT sound itself.

In a Polish study of Pawlaczyk et al. [21] with cross-sectional design, with 517 participants and a response rate of 78%, WT sound levels were calculated and randomly validated by measurement at location. Noise annoyance was measured using the 5-point ISO standard scale. Residential satisfaction, visual aspects, demographics and attitude towards the WTs were included as key confounders. The percentage of highly annoyed (%HA) increased significantly with sound levels of 35 to 53 dB Lden, and with a negative attitude towards wind turbines. The %HA was significantly and reversely associated with distance to the nearest wind turbine. 

A study of Radun et al. [22] in Finland included 429 people and had a response rate of 57%. WT sound level was calculated and measured and categorized into four exposure groups (25 up to 46 dB Lden). One of the main outcomes was indoor and outdoor annoyance. Trust in authorities and operators, visibility, economic benefits, age, gender, education, type of dwelling, and distance were adjusted for in the analysis. Sound level was significantly associated with the percentage highly annoyed (%HA) outdoor with an odds ratio (OR) of 1.41. No association with annoyance indoors was confirmed.

The cross-sectional study in China by Song et al. [23] included 227 participants living close to a wind farm (response rate 77%). Sound level was measured and categorized into 5 sound level classes (<40 dB up to >47.5 dB LAeq). Gender, age, residence time, visibility, NS, attitude, and general opinion about WTs were included as key confounders. The %HA increased with sound level from 39.5% to 75.0%. 

The Health Canada’s Community Noise and Health Study (CNHS) on the impact of wind turbines was featured in our 2017 review [1]. Since 2017, a first paper was published on aggregate annoyance from WTs, taking non-acoustic aspects into account [24]. The aggregate annoyance construct [24] accounted for annoyance from multiple WT features: noise, blinking warning lights, vibrations, visual impact and shadow flicker, explaining 58–69% of the variability in total annoyance. The association with distance to the turbines was confirmed in two large samples. Annoyance significantly increased in areas between 1 km and 550 m and was highest within 550 m.

In the next paper [25], the association of this aggregated annoyance index and a range of health complaints (high blood pressure and cortisol levels) and symptoms (dizziness or headache, and quality of life) were studied. Aggregate annoyance differed significantly between people reporting one or more symptoms (mean score 2.53 to 3.72) versus those without symptoms (0.96 to 1.41). No association with cortisol concentrations, systolic blood pressure, and rated quality of life was confirmed.

In their cross-sectional study, Botelho et al. [26] compared the role of WT sound to that of annoyance in the decisions people made about noise mitigating measures (36%). It was concluded that decisions to insulate the dwelling were directly related to WT sound levels and not to annoyance.

A cross-sectional Finnish study by Hongisto et al. [27] had, as their main aim, to derive an EEr for indoor annoyance from indoor sound due to large WTs (nominal electrical power of 3 to 5 MW). The association in 429 participants was consistent with those obtained for smaller WTs (sizes 0.15–3.0 MW) when the sound level was under 40 dB LAeq. Above 40 dB LAeq, the small number of participants prevented a reliable comparison to previous studies. At sound levels below 40 dB, the prevalence of high annoyance was less than 4%. The authors concluded that below 40 dB LAeq large WTs (>3 MW) leads to similar indoor noise annoyance levels as smaller ones (<1.5 MW) do.

In a listening experiment by Schäffer et al. [28], among 52 participants, stimuli were used representing different conditions of WT and other broadband sounds. The relative contributions of spectral shape, depth of periodic AM and random AM to short-term annoyance were tested. Confounding was not accounted for, but perceived loudness and perceived sound characteristics were included, and the ISO standard annoyance question adapted for acute effects. All three characteristics showed to affect annoyance comparable to an increase of up to 8 dB.

In a laboratory study by the same team [29] in 43 participants, WT sound level, amplitude modulation (AM) and visual aspects were linked to annoyance in 24 conditions combining visual in and auditory stimuli. Using a ‘within subject study design’, the same persons were tested in all conditions. Annoyance was measured using the 11-point ISO standard scale. Both visual and acoustical characteristics and attitude towards wind farms showed to affect noise annoyance. Important finding was that the initial visual setting strongly affected the annoyance ratings of subsequent conditions, hereby priming later reactions.

In the cross-sectional study of Haac et al. [30], 1043 participants (response rate 22%) were recruited both by telephone and online. Respondents were asked about audibility, annoyance, visual aspects, level of participation in local projects and personal characteristics as NS, attitudes and visual aspects of the wind farm. WT sound level was the most robust predictor of audibility and a weak, but significant, predictor of noise annoyance. Noise annoyance was best explained by visual disapproval (OR: 11.0; 95% CI: 4.8–25.4) and receiving personal benefits.

Hübner et al. [31] analysed a sample of surveys from the United States of America, Germany and Switzerland and included 1407 (USA) and 1015 (Germany and Switzerland) respondents with a response rate of 22%. An assessment scale including annoyance and symptoms of stress (NAS-Scale) was used. The NAS-Scale was negatively correlated with the perceptions of fairness of the wind project’s planning and development process. Distance to the nearest turbine and sound pressure levels were not significantly associated with annoyance, while NS and attitude towards planning fairness were.

In a longitudinal study among 212 (t1) and 133 (t2) subjects by Pohl et al. [32] annoyance was measured by a standard question (5-point ISO standard scale) and a stress index. The response rate and dropout rate were each 33%. The 104 non-responders were more often women (61%) and people without a view on a WT (62), whilst no difference in attitude was observed. Perceived WT sound was an important indicator of annoyance and stress, but association between distance and acceptance was not confirmed. The % of symptoms due to road sound (16% at both times) was higher than from WT sound (10%/7%).

In a qualitative study among 67 participants of Krogh et al. [33], 28 had abandoned their home because of the presence of a wind farm within 10 km; 31 were contemplating, 4 pre-emptively left their home before the wind farm started operating; and 4 had decided to remain. People with pre-existing medical conditions were concerned that living near a WT would have a negative effect on their symptoms and these worries affected their moving behaviour.

**Table 2:** Overview of the characteristics of the selected studies on WT sound and annoyance.

Author	Country	Design †	Sample Size (Response Rate) ‡	Quality	Exposure Type and Assessment	Outcome Type and Assessment	Confounders Considered in Analyses	Reported Associations
Klæboe and Sundfor, 2016 [18]	Norway	CS (after)	90 (38)	Moderate	WT sound pressure level (37–47 dBA LAeq)	Annoyance (ISO 5-point standard scale)	Attitudes, demographics, visual judgements, NS	Noise from WTs evaluated as 17–18 dBA more annoying than road traffic noise (within range of 11–26 dBA reported by [19,20]). Role of non-acoustical factors large
Pawlaczyk-Łuszczyń ska, 2018 [21]	Poland	CS	517 (78)	Moderate	WT calculated sound levels and randomly verified by in situ measurement A-weighted SPL (LAeq,T), A and G-weighted sound pressure levels (LCeq,T and LGeq,T)	Annoyance (ISO 5-point standard scale)	Satisfaction, visual aspects, demographics, attitude	%HA and WT sound level (OR > 1.00) and negative attitude towards WTs; decrease %HA with increasing distance (OR < 1.00),
Radun et al., 2019 [22]	Finland	CS	429 (57) 318 eligible for participation	High	WT A-weighted equivalent SPL, LAeq, and categorized [25–30], [30–35], [35–40] and [40–46]	Annoyance, self-reported sleep disturbance (indoor, outdoor)	Trust in authorities and operators, visibility, economic benefits, age, gender, education, type of dwelling, distance	WT sound level and annoyance outdoor OR 1.41 (1.14, 1.74) < 0.01 (R2 = 0.71) Indoor: none Sleep OR = 1.38 (1.16, 1.65) < 0.01(R2 = 0.50)
Song et al., 2016 [23]	China	CS	227 (77)	Moderate	WT A-weighted equivalent SPL, LAeq, sound levels (44.1–56.7 dBA)	Annoyance, Sleep disturbance (self-reported)	Gender, age residence time, visibility, NS, attitude, general opinion about WTs	%HA increased from 39.5% (95% CI: 28.4–51.4%) to 75.0% (95% CI: 50.9–91.3%. Sleep disturbance and LAeq r^2^ = 0.209
Michaud et al., 2018b [24]	Canada	CS	1238 (79)	High	WT calculated sound levels A- and C-weighted Distance; Blinking warning lights, vibrations, visual impact and shadow flicker	Integrated Annoyance score	Age, gender, education, lifestyle, chronic illness, stress, quality of life, dwelling characteristics,	Explained variance 58–69% Annoyance significantly increased in areas between 1 km and 550 m (mean 1.59; 95% CI 1.02, 2.15) and was highest within 550 m (mean 4.25; 95% CI 3.34, 5.16).
Michaud et al., 2018c [25]	Canada	CS	1238 (79)	High	Integrated Annoyance score	Blood pressure Cortisol levels), symptoms Quality of life Nonspecific	Age, gender, education, lifestyle, chronic illness, stress, quality of life, dwelling characteristics,	Total annoyance differed significantly between people reporting one or more symptoms (mean score 2.53 to 3.72) versus those without symptoms (0.96 to 1.41). No association with cortisol concentrations, systolic blood pressure, and rated quality of life was confirmed.
Botelho et al., 2017 [26]	Portugal	CS	80	Moderate	SPL LAeq	Annoyance, noise mitigating measures	Attitude, NS, visibility, co-ownership	Decisions to insulate house related to WT sound levels, not to annoyance.
Hongisto et al., 2017 [27]	Finland	CS	429 (55)	Moderate	Laeq modelled 26.7–44.2 dB LAeq	Annoyance (4-point scale)	Demographics, NS, residential satisfaction, attitude towards WTs, visibility of WTs, trust towards authorities or operators	Below 40 dB L_Aeq_ large WTs (>3 MW) lead to similar indoor noise annoyance levels as smaller ones (<1.5 MW) do
Haac et al., 2019 [30]	USA	CS	1043 (14–28)	Moderate	L1 hr max	Audibility, annoyance (not ISO standard)	Attitude, NS, moving into the area before or after the wind park was operationalized	Audibility annoyance: OR: 11.0; 95% CI: 4.8–25.4).
Schäffer et al., 2018 [28]	Switzerland	EXP	52	Moderate	AM, Laeq	Annoyance 11-point ISO standard scale	Perceived loudness, perceived sound characteristics	Effect of sound level, AM and visuals
Schäffer et al., 2019 [29]	Switzerland	EXP	43	High	WT sound (33–49 dBA) synthesized for distances 100–600 m, with and without periodic AM	Annoyance 11-point ISO standard scale	Gender, age, attitude towards WTs, NS and visual aspects	Increase in sound level and AM increased annoyance, presence of visualized landscape decreased annoyance, visibility of WT increased annoyance. Effect of attitude, not of other factors
Hübner et al., 2019 [31]	Germany/USA	CS	USA 900 (22%) Germany1029 (28%)	Moderate	Distance A-weighted LAeq-sound pressure level	Health symptoms, annoyance, stress, coping, sleep time, REM, self-reported disturbance	Range of confounders	Distance and SPL not correlated to noise annoyance; NS and attitude regarding fairness strongly associated with stress and annoyance
Pohl et al., 2018 [32]	Germany	LO	212/133 (Before/after 38%	Moderate	A-weighted LAeq sound pressure levels, recordings, distance	Annoyance (5-point ISO standard scale), stress	Attitude	Distance to closest WT (r = –0.13) and ISO SPL: r = 0.27) according to ISO 9613-2 (1993), r = 0.27).
Krogh et al., 2019 [33]	Canada	CS	67	Na	Distance	Tendency to move	Loss, grief, anxiety	Not mentioned

† Design: CS, cross-sectional study; LO, longitudinal; EXP, experiment; ‡: the number of people (N) and the response rate (in case of a cross-sectional study) (%).

#### 3.2.2. Sleep

The selected studies are listed in Table 3. In a case control field experiment, Lane et al. [11] used polysomnographic and diaries to measure sleep for five nights. Twenty-seven individuals (response rate 50%) participated, of whom 15 were from a WT exposed area. Distance to the nearest WT was used as indicator of exposure and sound levels were measured during the period of the experiment. Onset latency (SOL), wake after onset, total sleep time, time in bed, number of awakenings and sleep efficiency were included variables. Self-reported sleep disturbance was measured by the adapted Pittsburgh sleep quality index. No statistically significant differences were found between the two groups (self-reported and physiological/behavioural sleep measures) after adjustment for gender and age. 

A Danish cross-sectional study in 583,968 addresses [34] studied the association between modelled WT sound levels above 24 dB at the façade and low-frequency sound level indoor and the use of prescribed sleep medication. Age, gender, income, education, marital status, type of dwelling and distance to a nearby road were included as confounders. Results showed a weak association between a five-year averaged outdoor night-time WT sound level of >42 dBA and use of sleep medication OR of 1.14 (95% CI: 0.98, 1.33) per 10 dB increase. The association was strongest for the older age group.

A Finish cross-sectional study [22] among 429 people (response rate 57%) investigated the association between indoor WT sound levels and self-reported sleep. WT sound level was modelled and categorized in 5 dB groups in the range between 25 and 46 dB Lden. Trust in authorities and operators, visibility, economic benefits, age, gender, education, type of dwelling, and distance were adjusted for as important confounders. A significant but weak association between indoor sound level class and subjective sleep disturbance with an OR of 1.38 (95%CI: 1.16, 1.65) and Nagelkerke pseudo (R^2^ = 0.50) was found. Health concerns had more influence than WT sound level.

Morsing et al. [35] set up two laboratory experiments with six healthy students during three consecutive nights. Sound exposure consisted of recordings of WT sound with variations in sound pressure level, amplitude modulation (AM) strength and frequency, spectral content, turbine rotational frequency and beating behaviour. Sleep was measured by polysomnographic indicators and questionnaires. Heterogeneity between the two studies prevented firm conclusions regarding effects of WT sound on sleep.

A larger study [36], which was more representative than the study by Morsing et al. [35], exposed participants to recorded and more naturalistic WT sound. Participants were recruited from the general population aged 30–70, with a BMI below 30 kg/m^2^, habitual sleep times between 23:00 and 07:00 and a mean sleep duration of roughly eight hours. Exclusion criteria included the use of sleep medication, sleep apnoea and self-reported auditory acuity, confirmed during the first pilot night of the study. The total experiment lasted 3 nights with 1 habituation night, 1 night with realistic indoor WT sound exposure and 1 quiet night. Outcome measures included self-reported sleep quality and physiological measures. Key confounders were subjective stress and NS. Results showed that WT sound exposure affected the duration of REM sleep. No effects on other measured physiologic outcomes were detected. It was concluded that continuous WT sound with AM may impact sleep independent of habitual exposure to WT sound, whilst the habitually exposed group rated their sleep quality as low, reported more fatigue, and lower sleep quality, higher noise-induced sleep disturbance in the control and exposure night, compared to the reference group.

A Chinese study [23] among 227 participants (response rate 77%) included measured WT sound levels categorized into 5 sound levels (<40 dB up to >47.5 dB) and self-reported sleep disturbance. Gender, age, residence time, visibility, NS, attitude and general opinion about WTs were included as important confounders. The association between WT sound level and subjective sleep was significant but weak (Spearman corr. = 0.21).

The cross-sectional study of Kageyama et al. [37] in Japan among 1079 residents (response rate 47%) included field measurements on a limited set of addresses to estimate WT sound levels for each address. Self-reported sleep symptoms and insomnia were the main effects considered. Sound from road traffic, NS and attitudes towards WTs and demographics were adjusted for in analysis. No evidence was found for an adverse effect of WT sound on physical or mental health, self-reported sleep disturbance and insomnia. Insomnia was more prevalent in areas with WT sound levels above 40 dB LAeq at night. Sleep was primarily affected by NS and visual annoyance with WTs.

**Table 3:** Overview of the characteristics of the selected studies on WT sound and sleep (self-reported, physiological/behavioural).

Author	Country	Design †	Sample Size (Response Rate) ‡	Quality	Exposure Type and Assessment	Outcome Type and Assessment	Confounders Considered in Analyses	Reported Associations
Lane et al., 2016 [11]	Canada	CS	27	Low	Noise measurements in bedroom (LAeq and LAmax) for 5 consecutive nights	Actigraphy Subjective variables of sleep: Sleep diary	Age, gender	No statistically significant differences were found between the two groups on any of the objective and subjective sleep measures after adjustment for gender and age.
Poulsen et al., 2019 [34]	Denmark	CS	583,968 addresses after exclusion of people who emigrated	Moderate	A-weighted sound pressure level (10–10,000 Hz) outdoor and A weighted Low Frequency sound pressure level indoor (10–60 Hz)	Sleep (prescribed medication)	Age, gender, income, education, marital status Dwelling, distance to the road	Five-year mean outdoor night-time WT sound level of ≥42 dB was associated with a hazard ratio (HR) = 1.14 (95% CI: 0.98, 1.33) for sleep medication. Indoor night-time LF_WT among persons ≥ 65 exposed to ≥15 dB HR = 1.37 (0.81, 2.31) for sleep medication
Radun et al., 2019 [22]	Finland	CS	429 (57) 318 eligible	High	WT A-weighted equivalent SPL, LAeq, sound levels modelled and categorized [25–30], [30–35], [35–40] and [40–46]	Annoyance, self-reported sleep disturbance (in/outdoor)	Trust in authorities and operators, visibility, economic benefits, age, gender, education, type of dwelling, distance	Sound level and annoyance outdoor OR = 1.41 (1.14, 1.74) <0.01 (r^2^ = 0.71) Indoor: none Sleep 1.38 (1.16, 1.65) *p* < 0.01 (r^2^ = 0.50)
Morsing et al., 2018 [35]	Sweden	EXP	6	Low	AM, Frequency and beats predictors (taped)	Sleep (objective and subjective measures)	Age, gender	Chi^2^ 8–15 (subjective sleep disturbance Chi^2^ 7–11: Awakenings
Smith et al., 2019 [36]	Sweden	EXP	50	High	AM, Frequency and beats/slag predictors if bad sleep (taped)	Self-reported sleep quality and physiological measures	Subjective stress and NS	Longer REM sleep latency (+16.8 min) and lower amount of REM sleep (−11.1 min, −2.2%) in WTN nights. No effect on other measures.
Song et al., 2016 [23]	China	CS	227 (77)	Moderate	WT sound measurements, 5 noise level categories (44.1–56.7 dBA Lden)	Annoyance, Sleep disturbance (self-reported)	Gender, age residence time, visibility, NS, attitude, general opinion about WTs	%HA increased from 39.5% (95% CI: 28.4–51.4%) to 75.0% (95% CI: 50.9–91.3%. Sleep disturbance and LAeq r^2^ = 0.209
Kageyama et al., 2016 [37]	Japan	CS	1079 (47)	Moderate	LAeq,n outdoor, estimates per address 36–40 dBA and <35 dBA	Sleep symptoms, insomnia (both self-reported)	Road traffic, NS, attitude towards WTs, age, gender, education	Insomnia more prevalent in areas with levels > 40 at night

† Design: CS: cross-sectional study; LO: longitudinal; EXP: experiment; ‡: the number of people (N) and the response rate (in case of a cross-sectional study) (%).

#### 3.2.3. Cardiovascular Effects

The WHO evidence review on cardiovascular and metabolic effects [14] identified three cross-sectional studies investigating the association between WT sound and self-reported cardiovascular disease. An update to this review [6,7] identified three publications on two studies on the association between WT noise and hypertension: one cross-sectional study [25] and one cohort study [38]. No association between WT sound level and redemption of antihypertensive medication (indicator of hypertension) was found. 

Two new cohort studies investigated the association between WT noise and ischemic heart disease (IHD) [39,40]. A Danish cohort study [39] also investigated the association between WT sound level and the incidence of stroke. (n = 712,401). No conclusive evidence of an association between outdoor WT sound and IHD or stroke was confirmed, consistent with Bräuner et al. [41], providing little support to a causal association between outdoor long-term WT sound exposure and IHD. 

Bräuner et al. [42] concluded on “suggestive evidence” of an association between long-term exposure to WT sound and atrial fibrillation (AF) amongst female nurses. Of the 28,731 nurses involved in the cohort, 1413 developed AF. They were exposed to slightly higher levels of WT sound than the controls. A non-significant increased risk (95% CI: 1.05–1.61) of AF was found amongst nurses exposed to long-term (11-year running mean) indoor WT sound levels ≥ 20 dBA at night compared to nurses exposed to levels < 20 dBA. Further analysis of the results showed an increased, but insignificant risk of AF in the highest exposure group (>29.9 dB) based on a small proportion of the number of cases (2.8%). The relative risk of AF in the highest exposure group (6.3%) was higher than in the lower exposure groups (4.4%) but comparable to the relative risk in the non-exposed group (6.0%).

Analysis on the same cohort [41] did not identify strong evidence for an association between long-term WT sound exposure and stroke risk. 

In Table 4 the selected publications are listed.

#### 3.2.4. Metabolic Effects

In the WHO evidence review [14], three studies were identified regarding the association between night-time WT noise and self-reported incidence of diabetes [43,44,45]. No association was found between night-time WT sound level and risk of diabetes. The update published in 2020 [6,7] yielded two new studies investigating the association between WT noise and the incidence of diabetes (see Table 5): one cross-sectional study [46] and one cohort study [47], confirming earlier findings of no effect. No studies were identified that investigated the impact of WT noise on obesity.

#### 3.2.5. Mental Health and Cognitive Effects and Other Effects

The reviews of Clark and Paunovic [15,16] did not identify original studies on the association of WT sound with quality of life, wellbeing and mental health. Five systematic reviews on WT sound and on cognitive and mental health were selected. Due to study limitations, inconsistency and qualitative comparisons across studies, the authors conclude on low-quality evidence and no effect of WT sound on quality of life, wellbeing or mental health.

An update concerning cognitive and mental health effects and wellbeing, cancer, self-reported health and birth effects [17] showed additional evidence to conclude that there is low-quality evidence for an absence of effects of WT sound level on self-reported quality of life or health and low-quality evidence of an effect on mental disorders (anxiety, depression) and birth outcomes [48]. No evidence was confirmed of an effect on cancer. 

Freiberg et al. [9] also concluded no relationship between WT sound and stress effects and inconsistent findings concerning quality of life, and mental health problems (depression and anxiety).

### 3.3. Reviews on Social and Physical Aspects Other Than Noise

Two reviews on determinants of annoyance, one in general [8] and one on visual aspects [49] were identified. 

The narrative review by Simos et al. [8] (see Section 3.1), included 104 articles (on 67 studies) and discussed a range of determinants of annoyance, such as WT sound, infra- and low-frequency sound, shadow flicker, safety, landscape impacts and effects on real estate prices. At the outcome side, annoyance and the so-called WT syndrome were included. Shadow flicker had a weak association with annoyance and health indicators. Results show that community participation at an early phase can prevent negative perceptions associated with wind energy projects. While housing prices drop considerably first, these returned to normal after the park became operational.

In 2019 Freiberg et al. published a review [49] about the influence of visual aspects on annoyance and sleep disturbance next to acoustical aspects, identifying seventeen studies (2000–2018). The pooled prevalence of high annoyance due to visual aspects was 6% each. Results on other health effects were inconsistent, with evidence that WT visibility directly increases sleep disturbance. Other studies showed that annoyance by visibility, shadow flicker, and blinking lights was significantly and directly associated with sleep disturbance. An interaction effect of visual and auditory stimuli was confirmed in only one study.

### 3.4. Original Studies on Social and Physical Aspects Other Than Noise

The search identified 36 new articles on the effect of physical, social and personal factors on annoyance and other health effects. From these, 25 were selected for full text reading.

#### 3.4.1. Visual Aspects

Several studies investigated the visual aspects of WTs in relation to their acceptability [29,50,51,52,53,54,55] (see Table 6), by means of surveys, experiments, document analysis, and stakeholders’ and experts’ consultation, all aiming at mapping the role of visual aspects in the planning and decision process and at exploring ways to mitigate the negative environmental and social impacts of wind energy. 

The Schäffer [29] experiment was already described in Section 3.3.

Delicado et al. [50] analysed the media, environmental impact assessment reports and official positions on wind energy. Visual pollution is often brought forward as an important argument against wind farms, either as a risk of damaging the image of an area or as indicative of technological progress. Media analysis showed that the word “landscape” was hardly ever used, but the rural-urban divide came forward in opinion articles. Analysis of the EIA reports showed that objections against wind farms in view of landscape pollution was used most often by NGOs and in certain cases, by the tourism industry.

Grima-Murcia et al. [51] reported on a laboratory study among 14 respondents in which respondents were shown pictures of landscapes with different energy saving measures varying in duration of exposure. Effects were measured by means of questionnaires as well as electroencephalographic recordings (EEGs). No differences were found in EEG reactions between the different stimuli including WTs. Nuclear plants led to brain activity indicative of processing negative emotions in the 400 msec time frame, indicating that EEG recordings can be a useful procedure for measuring visual impact.

Lamy et al. [52] interviewed 15 residents living at varying distances from a wind farm. Visual impact was one of the main aspects influencing perception, next to economic benefits, safety issues, noise and renewable energy benefits.

In a survey among 474 adults by Frantal et al. [53] the influence of visual aspects of the landscape on the impact of wind farms was studied. The contribution was highly dependent on the local environmental and socioeconomic context, including noise annoyance, economic benefits and educational level.

A survey among 400 participants in four different central European countries [54] presented people with a range of pictures, with photoshopped WTs and indicators related to landscape planning (relief, land cover and landscape pattern). An objective method was aimed to predict the visual impact of onshore wind farms. None of the indices showed a significant association with the acceptability of the turbines.

Landeta-Manzano et al. [55] evaluated the intervention of a leading WT producer to safeguard acceptance of wind energy projects by local communities. This involved 47 stakeholders and 6 experts (*n* = 53) in a qualitative study using semi-structured interviews. The focus of interventions was on the visual impact of the developments, health and safety issues, community involvement and social investment in the community. With respect to visual impact, results of the consultation showed that lack of involvement in decisions on the location of the WT contributed most to community acceptance. 

**Table 6:** Overview of the characteristics of the selected studies on WT sound and visual aspects.

Author	Country	Design †	Quality	Sample Size †† (Response Rate)	Exposure Type and Assessment	Outcome Type and Assessment	Aspects Considered	Reported Associations
Schäffer et al., 2019 [29]	Switzerland	EXP	High	43	(i) Distance to wind turbine (ii) periodic AM (iii) visual setting.	Noise-annoyance rating (11-point ISO standard scale)	NS and visual aspects.	Presence of a visualised landscape decreased annoyance, visibility of WT increased annoyance
Delicado et al., 2017 [50]	Portugal	Media analysis	Moderate	Na	Media exposure.	Annoyance	Social aspects: acceptance and opinions. Visual and economic aspects.	Landscape matters are more visible and important and at times sufficient to reject approval or change of the siting of a wind farm.
Grima Murcia et al., 2017 [51]	Spain	LAB	Low	14	Visual stimuli and EEG	Amount of seconds a picture with or without wind turbines can be seen	Visual aspects	No effect
Lamy et al., 2017 [52]	USA	Qual	Low	15	Distance to wind turbines	Worry and concern about new projects	Visual impact, economics, noise and flicker effects, safety and personal experience with wind.	Economic benefits and visual aspects most important to participants, followed by noise, hazard to wildlife, and safety concerns.
Frantál et al., 2017 [53]	Czech Republic	CS	Moderate	474 (not mentioned)	(i) Distance to wind turbines, (ii) number of turbines, (iii) capacity and size turbines.	Annoyance	Visual aspects: landscape disruption	e.g., the percentage of participants who found visual disruption of the landscape the most noticeable negative impact.
Sklenicka and Zouhar, 2018 [54]	Czech Republic	CS	High	400 (not mentioned)	Link to the acceptance of wind farms by the public and authorities	Not mentioned. Participants were shown pictures including photoshopped wind turbines.	Visual aspects	Landscape indices, elevation landmarks, elevation variation. Visual impact is also quantified by using a formula.
Landeta-Manzano et al., 2018 [55]	Spain	LO Interviews	Moderate	153 (stake holders and experts)	(i) Main characteristic of the wind turbines. (ii) Frequency rates of sick leaves due to work accidents and Hazard Ratio with regard to the hours of exposure support of wind turbines	Health and safety issues, effects of noise and non-ionizing radiation, ‘perceived’ health risk linked to the level of community involvement	Social aspects: acceptance. Visual aspects, health and safety issues, community involvement and social investment.	Contribution to the community acceptance expressed in negative or positive scores and main actions in relation to safety in health.

† Design: CS: cross-sectional study, LO: longitudinal; EXP: experiment; Qual: qualitative study mixed methods; ††: the number of people (N) and the response rate (in case of a cross-sectional study) (%).

#### 3.4.2. Demographic, Personal, and Socioeconomic Factors

Next to visual aspects, it is known that demographics, personal, social and economic aspects also affect the level of annoyance from different noise sources [56,57,58]. Table 7 lists the studies selected for this review.

##### Demographics

Age, gender and educational level have not been identified as crucial predictors of noise annoyance in general. Usually, these variables are treated as confounders rather than as important determinants of annoyance. In the specific context of wind energy and WTs, there is evidence that gender [22] and educational level [30] play a role.

##### Personal Factors

Fear and NS have been identified as key predictors of annoyance and stress-related effects. NS refers to an internal state (physiological, psychological, attitude, lifestyle and activities) of a person increasing reactivity to sound in general. NS is partly genetic but can also be a consequence of a disease or anxiety disorder [59]. Several studies [18,22,25,29,36] reviewed in the previous sections, included NS and anxiety as confounders in their statistical analysis, confirming the independent role of NS on reaction to WTs. Anxiety and worry are primarily related to low frequency and infrasound and are assumed to be associated with the so-called vibroacoustic disease, a disorder not acknowledged in the medical world.

##### Social, Economic and Political Aspects

Evaluation of the consequences for quality of life is strongly rated to the social acceptance of WT projects by local communities. Next to sound, economic as well as social and political aspects determine acceptance and notions of fairness. The communication and relation between residents, local authorities and project developer is crucial. In the past 10 years, the relation between these different stakeholders seems to be increasingly polarized.

Many studies draw attention to the aspect of a fair planning process and local involvement and participation [60,61,62,63,64,65,66,67,68,69,70,71,72,73]. People are more willing to accept new turbines in their vicinity if they can participate in decision making, become co-owner of a wind park, and if the generated electricity is regionally consumed rather than exported. People newly exposed to WT sound are less willing to accept a wind farm than already exposed groups. Local circumstances should be adjusted for in a study on acceptance, and a complex set of individual and collective values should be considered, and the perspectives of scientists, policymakers and citizens should be integrated.

Clark and Botterill [68] show that different stakeholders raise different “facts” about health complaints. An example is the so-called WT syndrome, which is not generally accepted in the medical world and is grounds for a lot of debate.

Earlier studies concluded that economic aspects can also affect annoyance and co-ownership and benefits came forward as important predictors as compensating adverse responses to WT. A sense of control and benefit are important in this context [74]. 

More recent literature is focused on economic endpoints such as willingness to pay and to accept ET sound and the perceived reduction in housing values related to wind farms [75,76]. Thomson [75] concluded that people living close to a WT would be willing to pay for a windfarm to stay, whereas people living near a coal plant were willing to pay for the coal plant to be removed. This was not determined by demographics. Wen et al. [76], in contrast, concluded that respondents in different studies consistently showed increasing willingness to pay for moving wind farms to greater distances from their dwellings, depending on the number and height of WT, indicating a non-linear association.

**Table 7:** Overview of the characteristics of the selected studies on WT sound and physical, social and personal factors.

Author	Country	Design †	Quality	Sample Size (Response Rate) ††	Exposure Type and Assessment	Outcome Type and Assessment	Aspects Considered	Reported Associations
Clark and Botterill, 2018 [68]	Australia	Qual mixed	Low	22	Not mentioned	Health effects as a social phenomenon	Social and personal factors: wind turbine syndrome	Qualitative: stake, interest and legitimacy determine competing descriptions about the ‘facts’ of WT health effects.
Kongprasit et al., 2017 [67]	Thailand	CS	High	729 (93)	Public attitude and acceptance to wind farms	Percentage of respondents that work and live in or near the proposed site installations.	Social aspects	No significant differences regarding early brain processing when looking at landscapes with and without solar power systems or wind turbines.
Liebe et al., 2017 [63]	Switzerland	Panel, factorial survey, experiment (FSE)	High	1800 (Panel))	Public attitude and acceptance to wind farms	(i) Number of turbines (ii) electricity use (iii) distance to turbines.	Social aspects: acceptance and fairness	Overall acceptance levels in numbers of residents.
Kim et al., 2018 [70]	Republic of Korea	Interview, media and policy analysis	Low	7	Not mentioned	Not mentioned	Social aspects: acceptance.	Not mentioned.
Gölz and Wedderhoff, 2018 [71]	Germany	CS	Moderate	2009 (18)	Not mentioned.	Risk perception of energy system transformation.	Social aspects: acceptance, fairness and attitude	Structural equation model (SEM) using fit indices and descriptive data of regional acceptance figures.
Scherhaufer et al., 2017 [65]	Austria	Qual	Moderate	172	Not mentioned.	Not mentioned.	Social aspects: Acceptance. Economic aspect, NS, Visual aspects.	Patterns of acceptance and perceived importance defined as very to somewhat important.
Langer et al., 2018 [62]	Germany	CS	High	1400 (Panel)	(i) visibility from place of residence. (ii) Experience with wind energy. (iii) Number of WTs in vicinity, (iiii) Distance to place of residence.	Perceived side effects e.g., fear of infrasound.	Social aspects: acceptance. Visual aspects. Fear of infrasound.	Acceptance of wind turbines: Not affected by distance, Significant association with distributive justice Fear of Infrasound Mixed association with modes of participation
Scherhaufer et al., 2018 [72]	Austria	Qual	Moderate	241 (97/144)	Not mentioned	Not mentioned	Social aspects: acceptance.	Integrating scientific and lay people perspectives as a way forward.
Sæþórsdóttiret al., 2018 [73]	Iceland	CS	Moderate	1351	No link is made.	(i) Distance to wind turbines. (ii) Capacity and size of wind turbines. (iii) visibility of wind turbine. (iiii) Number of wind turbines.	Acceptance. Economic aspects: tourism and visual aspects for tourists.	One-third of the travellers is less likely to visit the Southern Highlands if a proposed wind farm were built, Two-thirds think that wind turbines would decrease the area’s attractiveness.
Wen et al., 2018 [76]	United Kingdom	Literature study: meta-analysis	Low	None.	(i) Distance toWTs (ii) Number of WTs (iii) WT height.	Preference for locating wind farms.	Visual aspects	Willingness To Accept and Willingness To Pay
Thomson and Kempton, 2018 [75]	United States	CS	Moderate	534 (34)	(i) Capacity of WTs (ii) ability to see and hear WTs. (iii) number of days WT is visible (iiii) cardinal direction from front of house.	Attitude towards wind turbines.	Visual aspects, auditory impacts and social aspects.	Amount of dollars (Willingness To Pay)

† Design: CS: cross-sectional study; LO: longitudinal; EXP: experiment; Qual: qualitative study mixed methods; ††: the number of people (N) and the response rate (in case of a cross-sectional study) (%).

### 3.5. Health-Related Effects of Low-Frequency Sound and Infrasound

Low-frequency sound and infrasound are attenuated less over larger distances and through building façades when compared to sound at higher frequencies. Low frequency (LF) sound includes sound at frequencies from about 100 Hz to 200 Hz. The range varies between countries and authors, e.g., 20–250 Hz [77], 10–160 Hz [78] and 8–100 Hz [79]. At frequencies above about 100 Hz it gradually merges into what we usually mean by ‘normal sound’: there is no clear boundary between the two sound ranges. Infrasound is the lowest part of LF sound and is defined by an upper boundary of 20 Hz. In this section, two questions are addressed concerning infrasound and LF sound: (1) Can inaudible infrasound cause a (health) effect? (2) Are the effects of infrasound and low-frequency sound different from normal sound?

The search in literature from 2017 yielded 24 publications, of which 15 papers were relevant for this update, two dating from before 2017. They included thirteen original experimental studies, one cross-sectional field study and one desktop study. We did not include reviews from this period as they did not provide new insights. Two experimental studies [80,81] were included after closing the search, resulting in 11 new studies as listed in Table 8.

#### 3.5.1. Original Studies on Audibility of Infrasound and Low-Frequency Sound

Low-frequency sound relates to road and air traffic and many other sources. LF sound in the 63 and 125 Hz octave bands (i.e., from 45 to 175 Hz) are usually included in calculation models and measurements of environmental sound. Infrasound is usually not included in modelling and measurements; it is produced by natural sources (e.g., wind and surf), transport and machinery at levels comparable to that of wind farms. Due to the high threshold of hearing, we are usually not aware of most of this infrasound.

Several studies reviewed here are part of the European EARS II project on infrasound, which was a follow-up of EARS I [82]. This first project concluded that below 20 Hz, the perception seems to change, and other sensory processes may give input to the auditory cortex. EARS II investigated brain activity in persons exposed to infrasound, including inaudible infrasound. An overview of results of the project was provided by Koch [83] and included the studies mentioned below.

Behler and Uppenkamp [84] investigated the loudness and unpleasantness of either 8 Hz or 32 Hz sound presented to 19 young, normal hearing persons. The maximum sound level was 140 dB. Brain activity was measured in persons exposed to the sounds of 1.5 s duration. Individual hearing threshold values were comparable to thresholds known from literature. The unpleasantness of each sound on average changed linearly with the perceived loudness, but individually, there were large variations. In an MRI-scanner, the sounds were presented at either low or medium loudness, depending on each person’s individual loudness scaling. At low loudness (5 out of the maximum 50 loudness units), activity in the auditory cortex was observed for both tones. At medium loudness (35 out of 50 loudness units), significant activity was observed. Sound is known to be processed in the auditory cortex and this study shows that this is also true for infrasound. Activation of the auditory cortex in this study was found to correlate better with perceived loudness than with the actual sound level.

Burke et al. [85] investigated whether audibility of one sound was influenced by the presence of another sound. This was tested in 13 young, normal hearing participants using two infrasound tones (5 Hz and 12 Hz), two tones at higher frequency (100 Hz and 1000 Hz), and pink noise between 250 and 4000 Hz. Individual hearing thresholds were measured three times and varied over a 5 dB range within participants and 20 dB or more between participants, consistent with the literature. Soft higher-frequency sound (5 dB over the threshold) did not significantly influence the detection threshold for the infrasound, while louder sound (50 dB over the threshold) led to a significant raise (1–9 dB) of the detection threshold of infrasound. In a third experiment, it was found that adding infrasound had no significant effect on the detection threshold of higher-frequency sound.

Weichenberger et al. [86] studied the effect of infrasound and low-frequency sound at discrete frequencies on brain activity. The hearing thresholds were determined for one ear in 14 young, normal hearing participants at eight frequencies ranging from 8 to 125 Hz and were all consistent with the literature. For each participant, a medium loud level was determined for 12 Hz infrasound. In an MRI-scanner, participants were either exposed to the 12 Hz tone, to the same tone 2 dB below the individual threshold, or to no sound, in each condition for 200 s. When exposed to the medium loud infrasound, no corresponding brain activity occurred, possibly due to adaptation (the brain response fades away). Over 200 s, average brain activation is not strong enough to appear in the measurements. In an earlier study [87], the same participants were exposed to short bursts (3 s) of 12 Hz at medium loudness, resulting in significant brain activity in the auditory cortex. Exposure to the 12 Hz infrasound at a level 2 dB below the individual hearing threshold elicited brain activity not found in the other conditions. Since activity occurred in the auditory cortex as well as brain areas associated with conflict regulation and emotional processing, this might indicate an unconscious reaction of the body. The authors speculate that, for prolonged exposure, there may be a ‘link’ with physiological and psychological health effects. 

Krahé et al. [81] performed an experimental study with participants exposed to four different levels of infrasound and to complete silence, each for half an hour, in a quiet, home-like room in a remote building. The level of infrasound was similar to standard thresholds used in Germany at three frequencies (3 Hz modulated, 5 and 10 Hz unmodulated) and 10 dB above this threshold at a frequency of 18 Hz. Results showed that, on average, the participants perceived the quiet period as not annoying, the period with lower frequencies (3 and 5 Hz) as somewhat annoying, with higher frequencies as moderately annoying. For most sounds (3, 10, 18 Hz), individual scores covered the entire scale from not annoying to very annoying. ‘Predisposed’ participants, who had had a problem with infrasound at their home, did not react differently from the other participants. The authors conclude that, essentially, perception is sensed by the ears, even when there is not always a clear hearing sensation.

Jurado and Marquardt [88] investigated the use of EEG in measuring perceived loudness of a very low-frequency sound. With a technique called frequency following response (FFR), electrodes on the head registered neural activity measured as a function of the loudness of the sound. In this way, the brain response to a constant sound of either 11 or 38 Hz was measured in 11 young, normal hearing participants. The general trend was that at zero loudness (sound at hearing threshold) the measured brain signal was close to the background of electric noise in the brain. On average, with increasing loudness, the signal increased and above a low to medium loudness remained constant. However, because of large individual differences, the authors concluded that the FFR signal did not correlate with the individual loudness perception and is not a useful method to measure loudness. 

Marquardt and Jurado [89] investigated the perception of amplitude modulation for two sounds at discrete frequencies (63 or 125 Hz) and either a modulation of these sounds at 8 Hz or an 8 Hz infrasound tone added to the unmodulated sound. The variation in amplitude of the modulated sound was 25% or 37.5% of the original tone amplitude. With a total of 400 samples, the sounds of each 1.2 s duration were played many times in random order and 12 normal hearing, young participants were asked to evaluate if the sample contained infrasound. The percentage of correct answers did not deviate from pure guessing. The authors concluded that a combination of a tone together with a constant 8 Hz infrasound resembles the tone amplitude modulated at 8 Hz (without the infrasound).

Jurado et al. [90] investigated if fluctuations in the level of a low-frequency sound influenced the perceived loudness of that sound. This was tested with 24 young, normal hearing participants who matched the loudness of three simple low-frequency tones (40, 63 or 80 Hz) and one 1000 Hz tone with a number of tone combinations. Each combination consisted of two tones close in frequency to one of the three simple tones, leading to fluctuations in amplitude at a frequency equal to the difference in the frequencies of both tones (being 1, 2, 5 and 12 Hz). Results showed that the effect of fluctuation at the lower frequencies on loudness was modest and corresponded to 2 dB or less; this agreed with loudness models described in the literature.

**Table 8:** Overview of the characteristics of the selected studies on WT low-frequency sound on audibility and response.

Author	Country	Design †	Participants ‡ (Age Range)	Exposure Type and Assessment	Outcome Type and Assessment	Reported Associations
Behler and Uppenkamp, 2020 [84]	Germany	LAB	19 (21–34)	8 or 32 Hz tones applied monaurally	Loudness and unpleasantness, brain activity	Same brain regions active as for typical audio sound: average in agreement with earlier studies, but large differences between subjects in rating sound
Burke et al., 2019 [85]	Germany	LAB	13 (18–30)	5 or 12 Hz tones and/or 100 or 1000 Hz or 250–4000 Hz pink noise applied monaurally	Detection threshold of one sound with or without other sound	Detection threshold of audio sound not influenced by IS tones; threshold of IS tones raised by medium loud audio sound
Weichenberger et al., 2017 [86]	Germany	LAB	14 (18–30)	8 tones in range 2.5–125 Hz applied monaurally	Spatial coherence and temporal independence in brain activity	No brain activity for medium-loud 12 Hz and for no sound; brain activity in AC and two other brain areas for near-threshold sound
Jurado and Marquardt, 2020a [88]	United Kingdom	LAB	13 (20–34)	soft to relatively loud 11 Hz or 38 Hz continuous sound (10–20 min)	Brain (EEG) response in relation to perceived loudness	No relevant effect of sound on EEG
Marquardt and Jurado, 2018 [89]	United Kingdom	LAB	12 (18–49)	8 Hz tone and 63 or 125 Hz tone, or 63/125 Hz tone modulated at 8 Hz with 25% or 37.5% modulation depth applied to preferred ear	Assessment: is sound amplitude modulated or sum of 8 and 63/125 Hz tone	Participants’ rating of each sound type not better than chance; for all sounds or slightly better
Jurado et al., 2019 [90]	Ecuador	LAB	34 (19–29)	4 tones (40, 63, 80 and 1000 Hz) and 4 two tone complexes centred at the same 4 frequencies, with frequency differences of 1, 2, 5 and 12 Hz.	Loudness matching of tone and tone complex	Results in agreement with literature and loudness models
van Kamp et al., 2017 [91]	Netherlands	CS	3972 (35)	calculated level of transport and industrial noise	Annoyance from humming noise, NS, residential satisfaction, house insulation	Lower background sound levels at night associated with higher annoyance from humming sounds
Maijala et al., 2020 [80,92]	Finland	CS, LAB	survey: 1351 (18–96);lab study: 26	wind turbine sound samples with highest infrasound levels and amplitude modulation values	Annoyance and physiological response	Participants who reported infrasound-related symptoms not able to perceive infrasound in noise samples and found samples with infrasound not more annoying than those without related symptoms
Jurado and Marquardt, 2020b [93]	United Kingdom	LAB	15 (20–34)	3 stimuli applied monaurally for 120 s: 6 ms 500 Hz bursts, 5 Hz repetition rate; 500 Hz modulated at 40 Hz 100% modulation depth; 4, 16 or 40 Hz	Myogenic (neck muscle) potential in response to vestibular stimulus	No significant vestibular response at 4 Hz and same for most subjects at 16 Hz
Conference papers + reports						
Takahashi, 2017 [94]	Japan	LAB	4 (21–47)	Infrasound stimuli at 16, 20, 25, 3.5, 40 and 50 Hz from speakers	Hearing thresholds (HT) and threshold values for unpleasantness (UT) and vibration in head (VhT)	UT higher than VhT, both higher than HT at all frequencies
Krahe et al., 2020 [81]	Germany	LAB	44 (-)	Infrasound stimuli (3, 5, 10, 18 Hz) above 85 dB(G) and silence	Perception, unpleasantness and physiological response (blood pressure, ECG, EEG, balance tests)	Perception of all sounds, predominantly by ear; no physiological effects; participants with earlier experience of infrasound not more sensitive

† Design: CS: cross-sectional study; LAB: laboratory study; ‡: the number of people (N) and age range.

#### 3.5.2. Effect of Lower Frequencies Compared to ‘Normal’ Sound

Infrasound and LF sound from WTs may affect the health of residents in a way other than how audio sound affects health. Effects as vibration of the body, nausea or dizziness, have been shown to occur in laboratory experiments but only at higher levels of infrasound than those from WTs. Here, we review studies into specific effects of infrasound and LF sound, WT related or not.

In a large survey, residents in three Dutch cities were asked if they were annoyed by a low frequency ‘humming noise, for example from ventilators’ [91]. Seven percent of the almost 4000 participants was highly annoyed by such noise. Other noise sources (road traffic, construction works, mopeds and neighbours) led to more highly annoyed persons, ranging from 13% to 22% of the participants. Several sources (rail traffic and industry) led to less annoyance (each about 4%). Persons dissatisfied with their residential situation as well as noise-sensitive persons reported more annoyance compared to people scoring high on residential satisfaction or low on NS. In the daytime, the percentage of persons highly annoyed by humming sounds was higher when background sound levels from road traffic were higher. At night, the reverse was true: a higher background level was related to somewhat less annoyance from humming sounds. No correlation was found between annoyance from humming sounds and sound insulation at the façade (double glazing, cavity wall filling, absorbing ventilation grille). 

Maijala et al. [80,92] describe a set of sub-studies investigating the role of infrasound in health complaints related to wind farms. A total of 70 out of 1351 survey respondents (5%) reported symptoms they attributed to infrasound from a wind farm. On average, these ‘symptomatic respondents’ lived closer to a wind farm than those without symptoms. Having chronic diseases, being annoyed by different aspects of WTs and considering WTs a health risk determined these symptoms. Ten percent of the participants considered WT infrasound a high risk to their personal health; eighteen percent as a high risk to health in general. Sound measurements were performed in two uninhabited dwellings at 1.5 km from a wind farm. Recordings with the highest infrasound and amplitude modulation (AM) levels were selected for the laboratory experiments. Results showed that (1) people reporting WT (infra)sound related symptoms did not exhibit an increased sensitivity for WT infrasound; (2) annoyance was related to total WT sound level and AM, not to infrasound; (3) WT infrasound or annoyance had no association with heart rate or heart rate variability, nor with skin conductance (as physiological measures of stress).

In the study of Krahé et al. [81], participants were submitted to several physiological tests during exposure. Tests involved blood pressure, heart rate and EEG. Tests showed no differences between the different exposures and no differences between predisposed participants and others.

#### 3.5.3. Sub-Audible Including Vestibular Effects

Vibroacoustic disease (VAD) and ‘visceral vibratory vestibular disease’ (VVVD), causing the WT syndrome (WTS), have been proposed as effects of WT sound exposure. No studies were published that support the existence of VAD or the VVVD. However, symptoms associated with WT sound can result from stress, possibly in relation to the presence of a wind farm.

The vestibular system can be activated by a loud mid- to high-frequency sound and Jurado and Marquardt [93] investigated if this was also true for infrasound. When activated, muscles in the neck and muscles attached to the eye contract, measured as an electromyogenic (EMG) reaction. In clinical practice, loud clicks are used, either from 6 millisecond sound bursts every 0.2 s or a continuous loud tone modulated at 40 Hz. The authors added three stimuli: a continuous sound over 120 s with a frequency of either 5, 16 or 40 Hz. The sounds were presented to 15 normal-hearing participants and to each ear separately, all at levels corresponding to loud sounds. Only the EMG reaction to vertical acceleration of the head was measured. The results showed that the 500 Hz sounds (as used in clinical tests) were significantly related to an EMG response for most participants. There was no significant response in one of both ears for five participants when using sound bursts and for five participants (one in both ears) using modulated sound. In contrast, at the low frequencies the response was predominantly not significant. At 4 Hz there was no significant response at all, at 16 and 40 Hz only in four of the 15 participants (of which one with both ears). The authors doubt if infrasound can produce an acceleration of the head at lower sound levels, such as occurring near WTs. 

Krahé et al. [81] submitted participants to tests concerning the sense of balance, including keeping balance, performing targeted movements, the occurrence of nystagmus (repetitive, uncontrolled eye movement) and eye fixation. Tests showed no differences between the different sound exposure scenarios (including silence) and no differences between predisposed participants and others.

#### 3.5.4. Effect of Vibrations

Takahashi [94] investigated if very low frequency/infrasound could be experienced as a vibration of the head or body. In an office type setting, four normal-hearing participants were exposed to six tones from 16 to 50 Hz. The hearing threshold was determined, as well as the levels in which the sound started to be ‘slightly annoying’, ‘very annoying’ or ‘too loud to work’. The levels in which the sound became unpleasant (unpleasant threshold) and in which the participants felt a ‘vibration in the head’ (vibration threshold) were determined. The results showed that the level where participants felt a vibration in the head was on average about 6 dB (at 16 Hz) to 15 dB (40 Hz) above their average hearing threshold. This vibration threshold almost coincided with levels at which the sound started to be slightly annoying. The threshold above which the sound was rated as unpleasant was still higher and was close to levels at which the sound started to be ‘too loud to work’. In an earlier study, Takahashi [95] also found that the head was the most sensitive part of the body to feel vibrations from infrasound.

Krahé et al. [81] asked participants to rate their perception of vibration, pressure and discomfort when exposed to four infrasounds or silence (see description in Section 3.5.1). In every sound scenario, including silence, all sensations were perceived mainly in the head area (head, brain, ears). Due to the low response and unequal numbers of participants, no conclusion could be drawn about the significance of differences between exposure scenarios.

## 4. Discussion

### 4.1. Summary of the Findings

Since 2017, the number, size and quality of relevant studies concerning WT sound and its effects on annoyance, sleep disturbance, and the cardiovascular and metabolic system has increased. In general, the new studies are population based, moderate to high-quality and have a larger geographical spread in comparison to the earlier evidence. Based on the new literature, we conclude on a robust association between the level of WT sound and annoyance. The percentage of highly annoyed residents increases when the sound level is higher, and the visual and aural intrusion explain a large part of the annoyance of residents. Other important predictors of annoyance are NS, attitudes towards WTs, health concerns and aspects related to the procedure and participation preceding the siting of a wind farm.

For other health effects such as sleep disturbance, insomnia, and cardiovascular and metabolic effects the findings are inconsistent. No relation was confirmed for metabolic effects (diabetes) and mental health. Studies on obesity and cognitive effects have not been performed. Earlier findings on the association between health symptoms and annoyance were confirmed in the new studies, but no conclusions can be drawn about the causal direction of this relation.

Although low-frequency sound and infrasound may have other effects other than ‘normal’ sound has, these effects are highly unlikely at sound levels typical for WTs. Brain studies show that LF and infrasound are processed in the same parts of the brain as ‘normal’ sound, and there is no evidence that sub-audible infrasound from WTs elicits any reaction. Acoustically, LF sound and infrasound differ from sound at higher frequencies: because of the low attenuation, low-frequency sound becomes relatively more important at larger distances and inside dwellings. Infrasound is attenuated even less, but coming from WTs, it is too weak for human perception at residential locations. 

### 4.2. Evidence on Adverse Health Effects of WT Sound

The evidence reviews on annoyance, sleep, cardiovascular and metabolic effects and cognition and mental health for the WHO [4,10,14,15,16,17] included WT sound. Together with high-quality reviews and updates, the earlier conclusions now have a more solid base.

Since 2017, several studies have been published on the association between WT sound and cardiovascular effects such as ischaemic heart disease, stroke, and medication used for hypertension. No significant effects were found. The so-called Danish Nurse cohort study reports, as worded by the authors, “suggestive evidence” for an association between long-term exposure to WT sound and atrial fibrillation (AF) amongst female nurses, possibly because of (chronic) annoyance. However, the association is non-significant.

The review on annoyance, sleep disturbance, cardiovascular and metabolic health outcomes [6,7] yielded two studies investigating the association between WT sound and the incidence of diabetes. Neither study found an association between WT sound and self-reported or diagnosed diabetes. There is also no evidence (no studies performed) of an association between WT sound and obesity.

For mental health and quality of life, there is insufficient evidence for a direct relation with WT sound level. Cognitive effects have not been studied in relation to WT sound. For neither low birth weight nor cancer (no studies), significant associations were found with WT sound.

Despite limited evidence, an exposure-effect relation was developed for annoyance from WT sound in the WHO Guidelines [96], and the related limit value of 45 dBA Lden was conditional. Meta-analysis based on the evidence since 2014 [6,7] allows for deriving a more solid EER for annoyance and possibly for sleep. The general exposure-effect relation for annoyance from WT sound includes all aspects that influence annoyance and thus averages over all local situations. The relation can therefore form an indication only of the annoyance levels to be expected in a local situation. One study shows that this relation can also be used for the more recent and larger (3 to 5 MW) turbines.

In an endeavour to develop an aggregated annoyance measure including annoyance from factors other than noise, Michaud et al. [24] have shown the complexity of annoyance due to WTs. Freiberg et al. [9] also recommend that studies should account for these complex pathways of annoyance that is influenced by different moderator and mediating variables.

### 4.3. Evidence on the Role of Physical, Social and Personal Factors

Noise sensitivity, attitude towards WTs, visual aspects and economic benefit appear as the most important mediators and moderators. Annoyance increases with amplitude modulation, and new evidence again shows an interaction with visual aspects. Landscape evaluation and other visual aspects show no evidence for a direct association. Participation in the decision-making process, co-ownership (literally and symbolically) and consumption of local energy appear as important determinants of acceptance. People are more willing to accept new turbines in their vicinity if they can participate in decision making, the turbines are owned by a group of citizens, and if the generated electricity is consumed in the region instead of being exported and in general experience a sense of control.

### 4.4. Evidence on Adverse Effects of Low-Frequency Sound and Infrasound

Except for one study, the studies published since 2017 show that infrasound and low-frequency sound is processed in the auditory cortex, where normal sound is processed. Moreover, hearing thresholds based on brain activity agree with those based on ‘classical’ psychoacoustics. The brain studies also show that very low-frequency sound, including infrasound, increases steeply in loudness when compared with normal sound, which again is known from ‘classical’ psychoacoustics. 

One study [86] suggests that infrasound of 12 Hz just below the threshold is associated with brain activity, although it is unclear what effect this can have elsewhere in the brain or body. The authors speculate that this may be linked to physiological as well as psychological health effects. Arguments against this hypothesis are given in [97]. In our opinion, we first need to be sure this is a true effect of an inaudible sound. The brain activity occurred near or at the audibility threshold and not at lower levels further away from the threshold, which would be necessary for wind farm infrasound to have an effect. Our conclusion is that it is necessary to study brain activation from infrasound at levels comparable to those near WTs or farms and with more realistic sounds before concluding that inaudible infrasound can influence residents.

The recent studies of possible effects of audible infrasound and low-frequency sound confirm earlier results [2]. When persons, including those complaining about WT infrasound, are exposed to WT sound, the total WT sound level and amplitude modulation may be a cause for increased annoyance, not the infrasound part. WT infrasound was found to have no effect on physiological measures of the autonomous nerve system (changes in heart rate, heart rate variability, skin conductance). Soft or inaudible infrasound or very low-frequency sound does not lead to a reaction of the vestibular system, at least not the part that detects vertical acceleration. When exposed to infrasound or very low-frequency sound, a vibration in the body or head is felt at sound levels close to or higher than the hearing threshold.

This leads to the conclusion that low-frequency sound is part of the total sound of WTs and has the same effects normal sound has; it can be annoying and may have effects on (getting to) sleep and, if chronic, this may lead to further health effects. This is also true for sound from other sources such as road, rail or air traffic. Because of the low attenuation, low-frequency sound becomes relatively more important at larger distances and inside dwellings. Infrasound is attenuated less, but coming from WTs and at typical distances to residences, it is too weak for human perception.

### 4.5. Strength and Limitations

Although an extensive grading system was not applied, the strength of this synthesis is the rigorous search and selection strategy used. This led to the identification of recent studies into the health effects of sound of WTs, including the effect of infrasound and low-frequency sound and mediating aspects as physical, social and personal factors. The limited means and time reduced the level of detail that could be extracted. The present scoping synthesis focused on studies investigating sleep disturbance, annoyance, cardiovascular and metabolic effects as primary outcome, and objectively measured or estimated noise levels as primary predictor. 

For the assessment of the quality of the reviews and studies concerning WT sound and health and physical, social and personal factors, we used the same criteria of the short and user-friendly instruments of the National Institute of Health [3]. Ratings are categorised as low, medium or high quality. 

### 4.6. Implication for Future Research

The conclusion in the WHO Guidelines [96] that studies on the health effects of WTs are of insufficient methodological, and statistical strength does not hold any longer. Since 2014, the number, study size and quality of the evidence on annoyance and sleep disturbance justify conducting a meta-analysis. For a range of clinical and mental health outcomes, evidence is increasing, but the number of studies is still too limited to perform such a meta-analysis.

New evidence about the association between WT sound and annoyance warrants closer examination of the studies regarding the feasibility to derive EErs (with the note that given the range of factors that influence WT noise annoyance, high-quality generalised EErs will not necessarily enable us to predict responses at the local level). For new evidence on the association between WT sound and objective sleep measures, a meta-analysis will be worthwhile considering. It is recommended to distinguish between physiological and self-reported sleep indicators, consistent with [10]. The new studies also provide more evidence on the role of the number of events and the L_max_ levels and it will be worthwhile to compare the outcomes from the different new studies, including these indicators. Although the associations between WT sound and cardiovascular and metabolic effects are weak, the new studies justify a closer look at quality and strength of evidence. A meta-analysis is not expected to be feasible. For the other effects, insufficient evidence is available to derive a relevant EEr. We note that, given the range of factors that influence WT noise annoyance and possibly further health effects, high-quality EErs will not lead to better predictions of effects in local projects.

## 5. Conclusions

With a level usually below 45 dB Lden, WT sound is modest when compared to other sources such as transportation (road, rail and air traffic) or industry. Nevertheless, at equal sound levels, sound from WTs is experienced as more annoying than that of many other sources. Living near a WT or hearing sound of WTs can lead to chronic annoyance among residents. For other health effects such as sleep disturbance, insomnia or mental health effects, the evidence is inconsistent or insufficient. There is no indication that the low-frequency component has other effects on residents other than normal sound nor that infrasound well below the hearing threshold can have any effect. The level and amplitude modulation of all WT sound are the main causes for increased annoyance, rather than low-frequency sound or infrasound.

There is evidence that sleep disturbance is associated with annoyance rather than to WT sound above a certain level. New evidence shows an association between total annoyance and health complaints, but we cannot draw conclusions about the direction of this relationship. The moderate effect of WT sound on annoyance and the range of factors predicting the levels of annoyance implies that reducing the impact of WT sound will profit from considering other aspects associated with annoyance. The relevance of factors such as participation in the planning process, procedural justice, feelings of fairness and balance of costs and benefits from WTs is strongly supported by current evidence. In summary: the health complaints are primarily associated with a range of contextual and personal factors rather than actual sound exposure levels.

## Figures and Tables

**Table 4:** (**a**) Overview of the characteristics of the selected studies on the association between WT sound and hypertension. (**b**) Overview of the characteristics of the selected studies on the association between WT sound and ischemic heart disease.

**(a)**							
**Author**	**Country**	**Design †**	**Study Population**			**Exposure Range (dB) in Lden**	Ascertainment Hypertension * **(prev/inc/mor)**
			**N (%) ‡**	**Gender #**	**Age Range (years)**		
Michaud et al., 2018c [25]	Canada	CS	1238 (79)	MF	18–79	<25, 25–30, 30–35, 35–40, 40–46	2 (prev)
Poulsen et al., 2018a [38]	Denmark	CO	535,675	MF	25–85	<24, 24–30, 30–36, 36–42, ≥42	3 (inc)
**(b)**							
**Author**	**Country**	**Design †**	**Study Population**			**Exposure Range (dB) in Lden**	Ascertainment IHD * **(prev/inc/mor)**
			**N (%) ‡**	**Gender #**	**Age Range (years)**		
Bräuner et al., 2018a [40]	Denmark	CO	23,994	F	≥44	Unexposed, <21.5, 21.5–25.4, 25.4–29.9, >29.9	3 (inc)
Poulsen et al., 2019b [39]	Denmark	CO	535,675	MF	25–85	<24, 24–30, 30–36, 36–42, ≥42	3 (inc)

† Design: CS: cross-sectional study; CO: cohort study; ‡: the number of people (N) and the response rate (in case of a cross-sectional study) (%); # M: men; F: females. * The method by which hypertension was ascertained: 1 = measurement of blood pressure levels or by means of a clinical interview, 2 = by means of a question as part of a questionnaire or interview (self-reported), 3 = by means of health registration database. Type of outcome: prev: prevalence; inc: incidence; mor: mortality.

**Table 5:** Overview of the characteristics of the selected studies on the association between WT sound and diabetes.

Author	Country	Design †	Study Population			Exposure Range (dB) in Lden	Ascertainment Diabetes ** (prev/inc/mor)
			N (%) ‡	Sex #	Age Range (years)		
Michaud et al., 2016b [46]	Canada	CS	1238 (79)	MF	18–79	<25, 25–30, 30–35, 35–40, 40–46	1, 2 (prev)
Poulsen et al., 2018a [47]	Denmark	CO	614,731	MF	25–85	<24, 24–<30, 30–<36, 36–<42, ≥42	3 (inc)

† Design: CS: cross-sectional study, CO: cohort study; ‡: the number of people (N) and the response rate (in case of a cross-sectional study) or attrition rate (in case of a cohort or case-control study) (%); # M: men, F: females; ** The method by which diabetes was ascertained: 1 = measurement/clinical interview, 2 = self-reported, 3 = healthcare registration. Type of outcome: prev: prevalence; inc: incidence; mor: mortality.

## Supplementary Materials

The following are available online at https://www.mdpi.com/article/10.3390/ijerph18179133/s1, S1: Key search terms for the period from 2017–2020, S2: Glossary.
