# Supplementary material for: Health Effects Related to Wind Turbine Sound: An Update
Source: Int J Environ Res Public Health. 2021 Aug 30;18(17):9133. doi: 10.3390/ijerph18179133 (PMC8430592; doi:10.3390/ijerph18179133)
Supplement: Supplementary file 1 [file ijerph-18-09133-s001.zip › ijerph-1345117-supplementary.pdf]

Supplementary Materials is provided in the Annexes : Annex 1: Search profiles Annex 2: Glossary .

S1: Key search terms for the period from 2017-2020

For the period up to July 2018 the same search strategy was followed as for our previous review [2] .

Table S1: Search Profiles per database and topic

**Search strategy in Scopus, July 2018-July 2020**
Topic: health effects of wind turbine sound
TITLE ("wind turbine*" OR "windmill*" OR "windmill*" OR "windfarm*" OR "wind farm*" OR "windpark*" OR "wind park*" OR "windenerg*" OR "wind energ*"))
AND (TITLE ("health effect*" OR "health risk*" OR "stress" OR "annoy*" OR "health impact*" OR "sleep" OR "noise avoid*" OR "noise abat*" OR "preval*" OR "inciden*" OR "adverse" OR "human health*" OR "avers*" OR "attitud*" OR "percept*" OR "perceiv*" OR "quality of life" OR "well being" OR "wellbeing" OR "concern*" OR "emot*" OR "accept*")) AND PUBYEAR > 2017
Topic: low frequency effects
TITLE("infrasound*" OR "low frequency nois*" OR "low frequency sound*" OR "infrasonic*" OR "low frequency thresh*" OR “audibi*”)
AND (TITLE("health effect*" OR "risk*" OR "stress" OR "annoy*" OR "health impact*" OR "sleep" OR "noise avoid*" OR "noise abat*" OR "preval*" OR "inciden*" OR "adverse" OR "human health*" OR "avers*" OR "attitud*" OR "percept*" OR "perceiv*" OR "quality of life" OR "well being" OR "wellbeing" OR "concern*" OR "emot*" OR "accept*"))
AND PUBYEAR > 2017
**Search strategy in Embase, July 2018-July 2020**
Topics: health effects of wind turbine sound and low frequency effects
#1. 'wind turbine'/exp OR 'wind farm'/exp OR 'wind turbine*':ti,ab OR 'wind farm*':ti,ab OR 'wind power'/exp
#2. 'low frequency noise'/exp OR (low:ti AND frequency:ti AND ('noise*':ti OR 'signal*':ti OR ‘noise':ti,ab OR 'low frequency sound':ti,ab OR 'low frequency ultrasound':ti,ab OR 'low frequency signal*':ti,ab OR 'low frequency thresh*':ti,ab OR 'infrasound'/exp OR 'infraso*':ti OR 'audability':ti,ab OR 'audibl*':ti
#3. 'noise'/exp/mj OR 'nois*':ti OR 'noise pollution'/exp/mj
#4. 'hearing'/exp/mj
#5. 'sound'/exp/mj OR 'sound*':ti
#6. 'annoyance'/exp OR 'annoy*':ti
#7. 'wellbeing'/exp OR 'health*':ti OR 'health'/exp OR 'health status'/exp OR 'wellbeing*':ti
#8. 'aversion*':ti OR 'stress*':ti OR 'complain*':ti OR 'distress*':ti OR 'disturb*':ti OR 'worries':ti OR (('sensiti*' NEAR/3 noise):ti) OR 'sound pressure level*':ti OR 'sleep disturbance*':ti OR 'sleep quality':ti OR 'stress'/exp OR 'cognitive*':ti OR 'aversion'/exp OR 'distress syndrome'/exp OR 'sleep quality'/exp OR 'perception*':ti OR 'unpleasant*':ti
#9. 'quality of life'/exp OR ('quality':ti AND ('life':ti OR living:ti))
#10. #2 OR #3 OR #4 OR #5 OR #6 OR #7 OR #8 OR #9
#11. #6 OR #7 OR #8 OR #9
#12. #1 AND #10
#13. #1 AND #10 AND [2017-2020]/py
#14. #1 AND #10 AND [2017-2020]/py AND ([dutch]/lim OR [english]/lim OR [french]/lim OR [german]/lim)
#15. #2 AND #11 NOT #1
#16. #2 AND #11 NOT #1 AND [2017-2020]/py

##17. #2 AND #11 NOT #1 AND [2017-2020]/py AND ([dutch]/lim OR [english]/lim OR [french]/lim OR [german]/lim)
**Search strategy in Psycinfo, Januari 2017-July 2020**
Topic: health effects of wind turbine sound
(("health effect*" or "risk*" or "stress*" or "annoy*" or "health impact*" or "sleep*" or "noise avoid*" or "noise abat*" or "preval*" or "inciden*" or "adverse" or "human health*" or "avers*" or "attitud*" or "percept*" or "perceiv*" or "quality of life" or "well being" or "wellbeing" or "concern*" or "emot*" or "accept*" or "commun*" or "engag*" or "activis*" or "prefer*").m_titl.) and (("wind turbine*" or "wind farm*" or "wind park*" or "windfarm*" or "windpark*" or "windmill*" or "wind mill*" or "wind energ*" or "windenerg*").m_titl.)
Topic: low frequency effects
("health effect*" or "risk*" or "stress" or "annoy*" or "health impact*" or "sleep*" or "noise avoid*" or "noise abat*" or "preval*" or "inciden*" or "adverse" or "human health*" or "avers*" or "attitud*" or "percept*" or "perceiv*" or "quality of life" or "well being" or "wellbeing" or "concern*" or "emot*" or "accept*" or "commun*" or "engag*" or "activis*" or "prefer*").m_titl. and ("infrasound*" or "low frequency nois*" or "low frequency sound*" or "infrasonic*" or "low frequency thresh*" or "audibi").m_titl. --

S2: Glossary

ACC	Anterior cingulate cortex
AM	Amplitude Modulation
Atrial fibrillation	Irregular and often rapid heart rate
BMI	Body Mass Index
CI	Confidence interval
CNHS	Canadian Noise and Health study
Cross-sectional	A study at one specific point in time
CTL	Community Tolerance Level
DALY	Disability-adjusted life year
dB	deciBel, a measure for the level of sound
dBA	A-weighted deciBel, corrected for human hearing
DEFRA	U.K. Department for Environment, Food & Rural Affairs
DEN	Day-evening-night equivalent level
EEG	Electroencephalographics (recordings)
EER	Exposure Effect Relation
EMG	Electromygram
Et al	denotes the co-authors of a publication
EU	European Union
FFR	Frequency Following Response
(%)HA	(percentage) Highly annoyed people
(%)HSD	(percentage) Highly Sleep Disturbed people
IHD	Ischeamic Heart Disease
Incidence	Measure of the probability of occurrence of a given (medical) condition in a population within a specific period of time
L_{den}	Day-evening-night equivalent sound level
L_{dn}	Day-night equivalent sound level
L_{Aeq}	A-weighted equivalent sound pressure level averaged over a period of time
L_{night}	Nighttime equivalent sound level
MW	MegaWatt (million Watt)
NAS	Noise Annoyance Stress
NS	Noise sensitivity
OR	Odds ratio
Polysomnography	A test used to diagnose sleep disorders.
Prevalence	Actual number of cases of disease or injury present in a population at any particular moment in time
PRISMA	Preferred Reporting Items for Systematic reviews and Meta-Analyses
REM	Rapid eye movement (sleep stage)
SD	Standard deviation
SPL	Sound Pressure Level: the actual sound level in certain conditions or at a certain time
VAD	Vibroacoustic disease
VVVD	Visceral vibratory vestibular disease
WHO	World Health Organization
WT	Wind Turbine
WTA	Willingness to Accept
WTP	Willingness to pay
